# Highlights of the 31st annual meeting of the Society for Immunotherapy of Cancer (SITC), 2016

**DOI:** 10.1186/s40425-017-0262-1

**Published:** 2017-07-18

**Authors:** James L. Gulley, Elizabeth A. Repasky, Laura S. Wood, Lisa H. Butterfield

**Affiliations:** 1 Genitourinary Malignancies Branch, Center for Cancer Research, NCI, 10 Center Dr., 13N240, Bethesda, MD 20892 USA; 2 Department of Immunology/CGP-L5-321, Roswell Park Cancer Institute, Elm & Carlton Streets, Buffalo, NY 14263 USA; 3 Cleveland Clinic Taussig Cancer Institute, 13907, Blackberry Circle, Strongsville, OH 44136 USA; 4 Department of Medicine, Surgery and Immunology, University of Pittsburgh Cancer Institute, 5117 Centre Avenue, Pittsburgh, PA 15213 USA

**Keywords:** Cancer, Immunotherapy, Checkpoint inhibitors, Combinations, Adoptive cellular therapy, Cancer vaccines, Microbiome, Tumor microenvironment, Bispecific antibodies, CAR T cells

## Abstract

Therapeutic efforts to engage the immune system against cancer have yielded exciting breakthroughs and a growing list of approved immune-based agents across a variety of disease states. Despite the early successes and durable responses associated with treatments such as immune checkpoint inhibition, there is still progress to be made in the field of cancer immunotherapy. The 31st annual meeting of the Society for Immunotherapy of Cancer (SITC 2016), which took place November 11–13, 2016 in National Harbor, Maryland, showcased the latest advancements in basic, translational, and clinical research focused on cancer immunology and immunotherapy. Novel therapeutic targets, insights into the dynamic tumor microenvironment, potential biomarkers, and novel combination approaches were some of the main themes covered at SITC 2016. This report summarizes key data and highlights from each session.

## Background

**Fig. 1:**
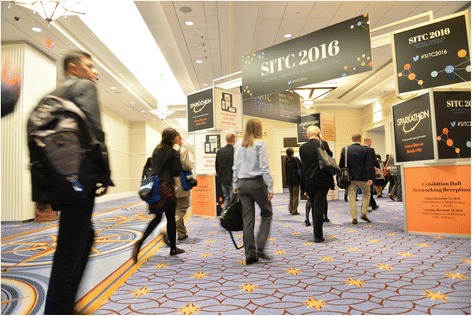
Exhibit Hall at the 31st Annual SITC Meeting in National Harbor, Maryland

The 31st annual meeting of the Society for Immunotherapy of Cancer (SITC 2016) was organized by Lisa H. Butterfield, PhD (University of Pittsburgh), James L. Gulley, MD, PhD, FACP (National Cancer Institutes, National Institutes of Health), Elizabeth A. Repasky, PhD (Roswell Park Cancer Institute), and Laura S. Wood, RN, MSN, OCN (Cleveland Clinic Taussig Cancer Institute). The attendance and international appeal of SITC’s annual meeting continues to surpass each previous year, with SITC 2016 welcoming over 2700 registered participants from 35 different countries. National and international groups presented the latest data from clinical and preclinical immunotherapy studies, provided updates on key organizational initiatives, and led discussions about the tumor microenvironment, combination immunotherapy approaches, current areas of challenge and opportunity in the field of cancer immunotherapy, and more (Fig. 1).

Outgoing SITC president, Dr. Howard L. Kaufman, MD, FACS (Rutgers Cancer Institute of New Jersey) opened SITC 2016 by remarking on the unprecedented growth experienced by the society in the past year. Indeed, SITC’s ability to draw a membership that spans academia, government representatives, industry leaders, and patient advocacy groups continued with the addition of the new membership category for nurses and advanced practitioners in 2016. Tragically, 2016 also marked the loss of noted physician scientist and program organizer, Holbrook E. Kohrt, MD, PhD, whose life and many contributions to the field of cancer immunotherapy were honored in a tribute to his memory at SITC 2016. In this report, we summarize the meeting, including updates on major initiatives and cutting-edge data from late-breaking abstracts. Slides and video of many presentations are available to SITC members and meeting attendees on the SITC website at http://sitc.sitcancer.org/2016/.

### Keynote address

Selected by the organizing committee for his seminal work in cellular biology, immunology, and extensive experience in cancer immunotherapy, Ira Mellman, PhD (Genentech), provided the Keynote Address on the mechanistic basis of cancer immunotherapy. Dr. Mellman began by presenting an overview of the “cancer immunity cycle”, highlighting cytotoxic T lymphocyte-associated protein 4 (CTLA-4) and anti-PD-1/programmed death-ligand 1 (PD-L1) as negative regulators of the T cell response that are necessary to maintain immune homeostasis [1]. Specifically, the PD-1/PD-L1 interaction inhibits T cell activation and attenuates effector function. As such, tumors and surrounding cells upregulate PD-L1 in response to T cell activity. Blocking the PD-1/PD-L1 pathway prevents or reverses T cell exhaustion and has broad antitumor activity in human cancers. In an effort to elucidate the mechanisms associated with response, PD-L1 expression was identified as a biomarker that could be used to stratify response to atezolizumab in bladder cancer [2]. Data from the randomized, phase III OAK study in lung cancer also illustrated that PD-L1 can enrich for responders, but PD-L1-negative patients still show benefit from atezolizumab therapy [3]. These findings suggest that the effects of PD-1/PD-L1 inhibitors extend beyond overcoming T cell exhaustion in the tumor bed and highlight the importance of identifying additional biomarkers that can identify responders or non-responders.

Exploring the downstream signaling effects of the PD-1/PD-L1 interaction, a liposome-based fluorescence energy resonance transfer (FRET) quenching assay, along with dephosphorylation experiments, determined that Shp2 binds with high selectivity to PD-1 to preferentially down regulate signaling via the CD28 costimulatory pathway, as opposed to signaling via the T cell receptor (TCR). Subsequently, it was shown that B7-dependent CD28 signaling is required to rescue exhausted CD8+ T cells by anti-PD-L1 in vivo. These results illustrate that the PD-L1/PD-1 interaction accelerates T cell exhaustion and restricts T cell priming or expansion; blocking this interaction using agents such as atezolizumab may facilitate T cell priming/expansion and block or reverse exhaustion.

Dr. Mellman concluded his presentation by discussing combinations of targeted agents and anti-PD-L1 therapy. In large screening studies, cobimetinib, a MEK inhibitor, showed efficacy in combination with PD-L1 inhibition despite evidence that MEK inhibition blocked T cell priming [4]. Exploring the mechanisms behind this synergy, an active MAP kinase pathway was found to be necessary only for naïve T cell expansion and differentiation into memory cells. In combination with anti-PD-L1, MEK inhibition protected tumor infiltrating CD8+ T cells from death driven by chronic TCR signaling; conceivably, this is the same pathway that induces T cell exhaustion. In a phase Ib trial, combination cobimetinib/atezolizumab led to objective or partial response in 20–25% of patients with colon cancer. Biomarker studies from this trial also illustrated that cobimetinib/atezolizumab increased CD8+ T cell infiltration in tumor samples. MEK and PD-L1 combined inhibition may act by preventing rather than reversing T cell exhaustion [5].

### Late-breaking abstracts

Five late-breaking abstracts representing novel cutting-edge data were selected for oral presentations. In the first, John Hunter, PhD (Compugen Inc.) explained how an international Compugen team used proprietary computational algorithms to identify a potential new T cell checkpoint, PVRIG, a member of the TIGIT molecular family that is expressed on T cells and NK cells and upregulated in human and murine tumors. The group then developed a high affinity antibody, COM701, which enhanced CD4+ and CD8+ T cell proliferation in vitro. In subsequent studies using a CT26 mouse model of colorectal cancer, PVRIG blockade combined with anti-PD-L1 therapy significantly reduced tumor growth (*p* = 0.0005; 56% tumor growth inhibition) to a higher degree than either agent alone, thus demonstrating the potential value of therapeutically targeting PVRG in addition to other B7 family checkpoints in the setting of malignancy.

Sonja Althammer, PhD (Definiens AG) addressed the prognostic potential of CD8+ and PD-L1+ tumor cell densities in determining response to anti-PD-L1 therapy (durvalumab). Automated image analysis of cell density in non-small cell lung cancer (NSCLC) tumor samples subsequently treated with durvalumab (*n* = 163; 77% patients previously treated) showed that high baseline combined CD8+/PD-L1+ cell densities (*n* = 26) were associated with higher overall response rate (ORR = 42%; 95% confidence interval [CI]: 23, 63) than low combined densities (ORR = 7%, 95% CI: 2, 17). A high proportion of combined CD8+/PD-L1+ cell densities was also associated with longer overall survival (OS; median OS = 24.3 months; 95% CI: 14.5, not reached [NR]), and progression-free survival (PFS; median PFS = 7.3 months; 95% CI: 4.0, 7.9) compared to high density of CD8+ cells (median OS = 17.8 months; 95% CI: 14.0, NR; median PFS = 5.3 months; 95% CI: 3.1, 7.4) or high PD-L1 status (TC+ ≥25%; median OS = 17.1 months; 95% CI: 9.8, 25.3; median PFS = 3.6 months; 95% CI: 2.6, 5.3) alone.

In the third late-breaking abstract session, Joaquim Bellmunt, MD, PhD (Dana-Farber/Brigham and Women’s Cancer Center) discussed highly anticipated data from the phase III KEYNOTE-045 trial of pembrolizumab versus investigators’ choice of standard chemotherapy (paclitaxel, docetaxel, or vinflunine), for advanced urothelial carcinoma (NCT02256436). Patients were enrolled regardless of PD-L1 status. This international study of 542 patients from 29 countries reported significantly longer OS in patients receiving pembrolizumab (HR 0.73; median 10.3 vs. 7.4 months; *p* = 0.0022) irrespective of PD-L1 expression. Pembrolizumab was also associated with fewer any-grade treatment-related AE compared to chemotherapy (60.9% vs. 90.2%). This trial was halted prematurely due to the markedly superior survival benefit in patients treated with pembrolizumab.

Preliminary data from an early phase study of a first-in-class antibody, lirilumab, which blocks inhibitory killer-cell immunoglobulin-like receptors (KIR) on NK cells (NCT01714739) were presented by Rom Leidner, MD (Earle A. Chiles Research Institute, Providence Cancer Center). This study investigated combination lirilumab plus nivolumab therapy in checkpoint inhibitor-naïve patients with squamous cell carcinoma of the head and neck (HNSCC) that progressed after platinum-based chemotherapy. Of the evaluable patients, 7/29 (24%) had an objective response per RECIST v1.1 criteria. Target tumor size decreased by >80% in 5/29 (17%) patients and median duration of response has not yet been reached. The lirilumab plus nivolumab combination demonstrated a manageable safety profile similar to that observed with nivolumab monotherapy. Further evaluation of this novel combination targeting two inhibitory pathways in NK cells and effector T cells is ongoing.

Finally, Padmanee Sharma, MD, PhD (University of Texas MD Anderson Cancer Center) discussed the first interim efficacy and safety results of the phase I/II CheckMate 032 study. The data presented were from two different dose schedules of ipilimumab and nivolumab (1 mg/3 mg vs. 3 mg/1 mg), versus nivolumab alone, in the open-label multicenter phase I/II trial for patients with advanced or metastatic urothelial cancer who progressed after platinum-based chemotherapy (NCT01928394). Preliminary results were very encouraging: ORR in the nivo 1/ipi 3 arm was 38.5%, compared with 26.0%, and 25.5% in the nivo 3/ipi 1 and nivolumab monotherapy arms, respectively. Median OS (months [95% CI]) was also higher in the nivo 1/ipi 3 group (10.2 [4.5, NR]) than the nivo 3/ipi 1 group (7.3 [5.6–11.4]). Side effects in the combination treatment groups were in line with other studies, with 30.8% of nivo 1/ipi 3 patients and 31.7% of nivo 3/ipi 1 patients experiencing a grade 3–4 treatment-related AE. Enrollment is ongoing.

### Update session: society initiatives

**Fig. 2:**
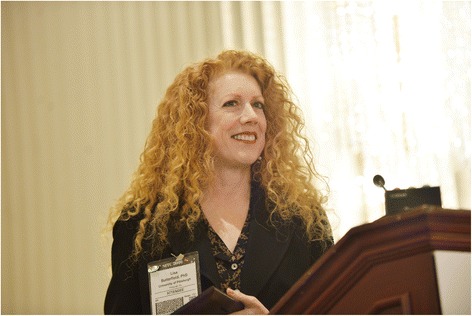
Incoming SITC President, Lisa H. Butterfield, PhD

In a session focused on SITC initiatives, incoming SITC President and Immune Biomarkers Task Force Chair Lisa Butterfield, PhD (University of Pittsburgh) presented recent activities undertaken by the SITC Immune Biomarkers Task Force (Fig. 2). Based on the success of previous workshops and publications, the SITC Immune Biomarkers Task Force reconvened to address progress and challenges in several key areas of biology that are only recently understood to impact the immune response: metabolism, the microbiome, and pathway signaling; new technologies and high-throughput approaches; novel and conventional agents affecting immunity; and bioinformatics, complex data analysis, and advances in biological sampling. From 2015 to 2016, four working groups (WG) collaborated to address the recent progress and challenges in each of these key areas. In this ongoing effort, these WG have generated five separate white papers and led a dedicated workshop, Immunotherapy Biomarkers 2016: Overcoming the Barriers, held in collaboration with the NIH. In addition, the WG members and others authored short reports highlighting novel technologies used for biomarker development in a series published in the *Journal for ImmunoTherapy of Cancer* (JITC).

Jérôme Galon, PhD (INSERM) presented the latest results of the SITC Immunoscore Validation Project. In an effort to validate the Immunoscore, a standardized immunohistochemistry-based assay to measure the immune contexture in and around tumors, SITC led an international, global collaborative effort to quantify tumor samples from patients with stage I-III colon cancer using the Immunoscore assay. Dr. Galon reported the final results of the study illustrating that the primary objective of the study was met: Immunoscore classification (high vs. low) predicted time-to-recurrence. In secondary analyses, a subgroup of high-risk, Immunoscore-low patients was identified in the stage II cohort. New data on microsatellite instability (MSI) status were also presented. These findings illustrate the prognostic value of the Immunoscore assay in colon cancer patients and justify the use of immune parameters as a new component of cancer classification.

### Update session: cancer immunotherapy trials network

In line with the goal of leading the design and conduct of cancer immunotherapy trials to expedite approval of promising agents, representatives from the Cancer Immunotherapy Trials Network (CITN) presented ten clinical trials of high-priority immunotherapy agents. Leading this session, Jeffrey Miller, MD (University of Minnesota) highlighted a phase II study of pembrolizumab in unresectable, recurrent advanced Merkel cell carcinoma (NCT02267603), which illustrated the highest response rates for an agent targeting programmed cell death protein 1 (PD-1) in any solid tumor to date. In addition, Dr. Miller highlighted a phase II trial of pembrolizumab in patients with relapsed or refractory stage IB-IVB mycosis fungoides or Sézary syndrome (NCT02243579), and three phase I studies: pembrolizumab in HIV-positive patients with relapsed/refractory malignancies (NCT02595866), neoadjuvant CD40 agonist alone or in combination with chemotherapy in patients with recently diagnosed resectable pancreatic carcinoma (NCT02588443), and a dose escalation study of subcutaneous recombinant IL-15 in advanced solid tumors (NCT01727076).

Continuing this session, Lawrence Fong, MD (University of California, San Francisco) reported results from an ongoing multi-institution trial (NCT01881867) testing sipuleucel-T in combination with subcutaneous IL-7. This study found that the combination therapy was generally well tolerated and decreased the neutrophil/lymphocyte ratio in circulation, suggesting that IL-7 may contribute to greater expansion of lymphocytes than sipuleucel-T alone. Kunle Odunsi, MD, PhD (Roswell Park Cancer Institute Center for Immunotherapy) presented a study (NCT02042430) designed to investigate the effects of indoleamine 2, 3-dioxygenase (IDO)-1 inhibition via oral INCB024360 on the tumor microenvironment (TME). In this study, patients experienced an increase in CD8+ T cell tumor infiltrate as well as a shift in the interferon (IFN) signature, and an increase in genes associated with natural killer (NK) cells and the Th1 subset. In the final presentation of this session, Steven Fling, PhD (Fred Hutchinson Cancer Research Center) presented data from a phase II randomized multi-center trial (NCT02129075) testing the effects of pre-treatment with the Flt3 ligand CDX-301 prior to vaccination with CDX-1401 (an antibody specific for DEC205 and NY-ESO-1). The addition of CDX-301 resulted in an increase in innate immune cells as well as significantly higher anti-NY-ESO-1 antibody titers and rapid NY-ESO-1-specific T cell responses. Moreover, the combination was well-tolerated, with no adverse events (AE) requiring discontinuation of treatment.

### Update session: government agencies

Kevin Howcroft, PhD (National Cancer Institute [NCI], National Institutes of Health [NIH]) began the government agencies update session with an overview of the NCI portfolio in cancer immunology and immunotherapy. Agents that directly modulate cancer immunity (e.g., cytokines, checkpoint inhibitors, vaccines, adoptive cellular therapy) were included in the analysis, but not antibodies or agents directed at cancer cell targets, or with actions mediated through signal transduction or cytotoxic payload (e.g., bevacizumab, trastuzumab, immunotoxin). An overview of funding mechanisms from the Center for Cancer Training, which supports training and career development, showed that 8% of awards and grants support immunotherapy initiatives. In addition, 6% of extramural grants from the Division of Cancer Biology (DCB) and 13% of grants from the Division of Cancer Treatment and Diagnosis (DCTD) support immunotherapy research. Moreover, the DCTD has also supported 95 immunotherapy clinical trials between 2010 and 2016, including phase III randomized trials for novel combinations, rare tumors, pediatric malignancies, and pilot studies to determine biological endpoints. In 2017, there will be funding announcements for multiple Cancer Immune Monitoring Analysis Centers and a single Cancer Immunotherapy Data Commons to provide centralized support for immunotherapy clinical trials.

In the second presentation of this session, Raj K. Puri, MD, PhD (U.S. Food and Drug Administration [FDA]) provided an overview of FDA regulatory updates related to cancer immunotherapy. Dr. Puri described the structure of the FDA and the various centers overseeing the development and approval of drugs and biologics. On June 29, 2016, FDA Commissioner, Dr. Robert Califf, announced the opening of the FDA’s Oncology Center of Excellence (OCE). The OCE will leverage the combined skills of regulatory scientists and reviewers with expertise in drugs, biologics, and devices to expedite the development of oncology therapies, in particular novel combinations. Dr. Puri highlighted guidance documents intended to move the immunotherapy field forward and summarized programs including Fast Track, Breakthrough Therapy, Accelerated Approval, and Priority Review that were developed to accelerate relevant therapies through approval. Dr. Puri emphasized the importance of the FDA’s collaborations with international agencies, including the European Medicines Agency, Health Canada, and the Japan Pharmaceuticals and Medical Devices Agency. He concluded by encouraging the audience to take advantage of the numerous resources available from the FDA.

### Tumor microenvironment

This session provided mechanistic insights into the suppressive nature of this unique environment, and suggestions for how this knowledge can be used to therapeutic effect. To investigate the role of neuropilin-1 (Nrp1) in the TME, Abigail E. Overacre-Delgoffe (University of Pittsburgh) utilized a model of melanoma in Nrp1L/LFoxP3Cre-YFP/DTR-GFP mice in which regulatory T cells (Treg) are either wild-type (WT; 50%) or Nrp1-deficient (50%). Treg lacking Nrp1 produced significantly more IFNγ than WT Treg, which led to functional impairment of adjacent WT Treg in the TME. The loss of suppressive function in WT Treg cells was sufficient to allow antitumor immune mechanisms to eradicate B16.F10 melanoma cells. Subsequent studies in human tissue demonstrated that Treg cells in peripheral blood, and within melanoma and HNSCC tissue samples, express Nrp1, which correlated with poor prognosis. The ability of human WT and Nrp1−/− Treg to suppress effector cells was significantly impaired when cultured with IFNγ, further supporting Nrp1 as a potential therapeutic target.

Work presented by Justin Kline, MD (University of Chicago) sought to understand how antigen-specific immune responses are mounted or blunted in systemic malignancy, as there are no dedicated tumor-draining lymph nodes (LN) to potentiate an effective immune response. Using a transplantable murine model of acute myeloid leukemia (AML) in which tumor antigen-specific CD8+ T cells undergo deletional tolerance, this study found that only the cross-presenting CD8α + CD11c + dendritic cells (DC) engulf AML-derived cellular material, and these professional antigen-presenting cells (APC) are required for antigen cross-presentation to CD8+ T cells in vitro. CD8α + DC were also found to be responsible for the systemic induction of CD8+ T cell tolerance in vivo. Investigators noted a striking difference in gene expression profiles between DC that had taken up cellular material from AML and those that had not. Activation of CD8α + DC with a toll-like receptor (TLR)3 agonist was sufficient to break AML-induced tolerance and allow CD8+ T cells to mount an effective antitumor response.

### State-of-the-art immunotherapies: challenges and opportunities

Nicola Annels, PhD (University of Surrey) presented phase I/II data on Coxsackievirus A21 (CVA21), a novel oncolytic virus targeted to ICAM-1, in non-muscle invasive bladder cancer (NIMBC). In the CANON study, patients with NIMBC, which has characteristic upregulation of ICAM-1 expression, received neo-adjuvant CVA21 or low dose mitomycin C plus CVA21 prior to surgical removal. Intravesicular CVA21 alone or in combination was well tolerated, with no grade 2 or higher treatment-related AE reported. Moreover, CVA21 demonstrated clinical activity through viral-induced surface hemorrhage and inflammation, as well as tumor-specific viral replication, with evidence of viral-induced apoptotic tumor cell death. Multispectral immunohistochemistry demonstrated increases in immune cell infiltration in NIMBC tissue. Gene expression analyses illustrated widespread increases in interferon-induced genes, viral RNA, and immune checkpoint genes. Urinary analysis indicated that 11/16 (69%) patients had increased levels of HMGB1, an important mediator of inflammation. These results demonstrate the tolerability of CVA21 and evidence for subsequent local and potential systemic antitumor immune responses, warranting further study of this novel oncolytic virus for bladder cancer.

A presentation by Andreas Lundqvist, PhD (Karolinska Institutet) focused on the potential of IL-15 to extend the antitumor activity of NK cells through mTOR-mediated metabolic processes. Compared with NK cells not exposed to IL-15, IL-15-treated NK cells maintained higher levels of activity with reduced levels of apoptosis, and a higher level of proliferation and cytotoxic activity when cultured with tumor cells or exposed to tumor supernatant. Tumor-derived prostaglandin-E2 suppressed IL-2 cultured NK cells, while IL-15- stimulated cells remain activated. Genome-wide expression analysis showed a correlation between mTOR signaling and genes related to cellular metabolism and respiration, which were blocked by mTOR inhibition. Moreover, mTOR-independent STAT-5 signaling contributed to improved NK cell function during cytokine activation but not during withdrawal. This study furthers understanding of the mechanisms regulating activation and maintenance of tumor-reactive NK cells and supports the use of IL-15 with adoptive NK cell-based therapies [6].

Cara Haymaker, PhD (MD Anderson Cancer Center) presented phase I/II data of intratumoral TLR9 agonist, IMO-2125 (4 mg to 32 mg dose escalation), alone or in combination with ipilimumab (3 mg/kg) in patients with anti-PD-1-refractory metastatic melanoma. At the time of data cut-off, data from 10 patients were available. There were no treatment discontinuations due to an AE in the combination group, and no treatment-related deaths. Early data showed a 30% (3/10) response rate, two patients with partial response, and one patient with an unconfirmed complete response. Flow cytometric analyses illustrated rapid maturation of the CD1c + CD303- myeloid DC 1 subset in the IMO-2125 injected tumor 24 h post-treatment compared to pre-treatment biopsies. Moreover, biopsies from responders demonstrated a higher rate of proliferation (Ki67 index) and activation of CD8+ T cells vs. pretreatment biopsies, and analysis of plasma indicated an increase in circulating IFNγ levels in responders. Further studies are underway to evaluate the role of IMO-2125 in combination with pembrolizumab after evidence of upregulation of PD-L1 in post-injection biopsies.

### Metabolic and age-associated dysregulation of anti-cancer immunity

In a discussion about metabolic dysregulation of anti-cancer immunity, Mads Hald Andersen, PhD (Herlev University Hospital) introduced the idea of generating T cells that target suppressive components of the TME, including Treg, PD-L1, and IDO. Such autoreactive T cells can be found in the peripheral blood of healthy donors and within the blood and tumors of patients with cancer [7–10]. Using a cancer vaccine approach, these T cells can be expanded and activated in vivo to kill cancer cells in an antigen-specific manner. This approach is actively being investigated as a monotherapy or in combination with other agents in early phase clinical trials for several different malignancies [11].

Dawn Bowdish, PhD (McMaster University) presented work about age-associated dysregulation of the myeloid-derived suppressor cell (MDSC) compartment that should be taken into consideration when designing immunotherapeutic approaches to cancer. The product of inappropriate myelopoiesis, MDSC are potent suppressors of T cell proliferation and are associated with poor outcomes in many models of cancer [12–15]. In addition, increased numbers of MDSC in the circulation are associated with metastasis [16, 17] and decreased responsiveness to immunotherapy [18, 19]. Importantly, MDSC increase with age and a past history of cancer correlates with this increase in MDSC numbers [20]. In the absence of cancer, chronic age-associated inflammation creates an environment replete with MDSC-promoting factors that causes premature egress of immature myeloid cells from the bone marrow. This phenomenon supports the integration of aging animals in preclinical studies and suggests that depletion of MDSC may increase the efficacy of immunotherapies.

### Promoting and measuring antitumor immunity

Lisa H. Butterfield, PhD (University of Pittsburgh) presented on her group’s work improving antitumor immunity using dendritic cell (DC)-based vaccine approaches in melanoma and hepatocellular carcinoma (HCC). Dr. Butterfield summarized results from phase I and II trials using autologous DC pulsed with melanoma antigen MART-1_27-35_ peptide, and transduced with an adenovirus encoding full-length MART-1. In these studies, patients with the best clinical outcomes had evidence of determinant spreading to other melanoma-associated antigens [21, 22]. In order to improve potential responses, a novel adenovirus encoding three full-length melanoma antigens was combined with an IFNα boost in a recent trial. In preliminary results, 2/11 patients with measureable disease had a partial response, while 7/11 had ongoing stable disease. Standardized IFN-γ ELISPOT assays demonstrated CD8+ and CD4+ T cell responses to target antigens and evidence of induced determinant spreading. Gene expression analysis, to investigate markers in blood and tumor samples, and measure immune checkpoint expression, is underway to further elucidate the mechanisms underlying antitumor immunity. In recent data from studies using alpha fetoprotein (AFP) peptide and protein DC vaccines in HCC, tumor-derived AFP had a negative impact on T cell proliferation, and gene expression arrays revealed that tumor AFP affected signaling pathways involved in lipid metabolism. Moreover, in tumor-derived AFP exposed DC there was a reduction in mitochondrial mass, number of active mitochondria, oxidative phosphorylation, and in the master regulator of mitochondrial biosynthesis, PGC1alpha. Thus, therapeutic approaches that antagonize the effects of tumor-derived AFP may be necessary to enhance antitumor immunity.

### Richard smalley, md memorial lectureship

**Fig. 3:**
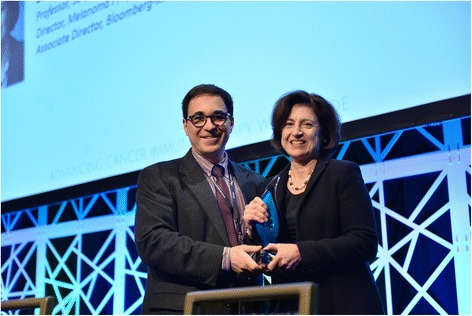
SITC President, Howard L. Kaufman, MD, FACS, presents Smalley Award to Suzanne L. Topalian, MD

The Richard V. Smalley, MD Memorial Award and Lectureship honors the memory and scientific legacy of past SITC president and charter member Dr. Richard V. Smalley and is presented annually to a distinguished leader whose research has made a significant contribution to advancing cancer immunotherapy, and has important clinical impact. The recipient of this year’s award was Suzanne L. Topalian, MD (Johns Hopkins University) (Fig. 3). Dr. Topalian’s work in antitumor immunity laid the foundation for the development of a number of immunotherapeutic modalities including cancer vaccines, adoptive T cell transfer, and immune-modulating monoclonal antibodies. In her keynote address titled “PD-1 Blockade in Cancer Treatment: Immunotherapy Meets Precision Medicine”, Dr. Topalian summarized work from the past several years regarding anti-PD-1 for the treatment of cancer, and addressed the need to develop biomarkers to better guide this therapy.

Dr. Topalian explained that the PD-1/L1 axis answered a longstanding question in the field of cancer immunology: what prevents cancer-specific T cells from eliminating tumors? Indeed, this important pathway has emerged as a mechanism that promotes local immune suppression within the TME in many solid tumors. Targeting this pathway therapeutically has led to durable remissions in a subset of patients across a variety of malignancies, hence the current challenge to enhance the impact of immunotherapy, in larger numbers of patients. Of principle interest is the development of biomarkers to identify patients or tumor types most likely to respond to treatment, and potentially to guide combination therapy. Such biomarkers will necessarily be complex and multifactorial, and patient-specific aspects such as T cell repertoire, T cell functional state, and the presence of immune-suppressive myeloid cells will need to be taken into account. Dr. Topalian closed her keynote address with the assertion that crucial battles in the war against cancer have already been won, and we now know what needs to be done in the laboratory and the clinic to finally win this war.

### Beyond single agents: the future of combination immunotherapy

This session sought to apply rationale and rigor to evaluate the overwhelming number of clinical trials of immunotherapy combinations. The first presentation was given by Ignacio Melero, MD, PhD (University of Navarra), who quipped that our current approach to combination therapy is akin to trying to win the lottery by buying all the tickets. Dr. Melero’s talk went on to emphasize the importance of choosing agents with complementary mechanisms of action capable of priming the immune system, removing co-inhibition, providing co-stimulation, and helping to condition the TME. Next, Alan J. Korman, PhD (Bristol-Myers Squibb) spoke about two strategies for improving the activity of anti-CTLA-4 immunotherapy, especially with respect to combination approaches. Strategies for next generation anti-CTLA-4 antibodies included non-fucosylated ipilimumab for enhanced activity via increased FcγR binding and a Probody™ version of anti-CTLA-4 for improved safety that localizes the drug activity to the tumor. Addressing the issue of patient selection for combination immunotherapy, Jérôme Galon, PhD (INSERM) spoke about the prognostic ability and potential of the Immunoscore, which is a histology-based assay to assess the immune contexture in and around tumors. Knowledge of the pre-existing antitumor immunity could guide efficient and personalized immunotherapy selection for patients.

The second part of the session on combination immunotherapy led with Drew M. Pardoll, MD, PhD (Johns Hopkins University) discussing the T cell repertoire as a biomarker and a means to guide precision immunotherapy. Similar to the relationship between tumor mutation load and response to treatment, analysis of TCR specificities could yield valuable prognostic information. In addition, the emergence of new technology such as mutation associated neoantigen functional expansion of specific T cells (MANAFEST) could help define antigenic peptides to formulate personalized vaccines. Next, Erminia Massarelli, MD, PhD, MS, (University of Texas MD Anderson Cancer Center) presented safety and efficacy data from studies of urelumab, an anti-CD137 monoclonal antibody that enhanced T and NK cell antitumor activity in preclinical models. The combination trial with nivolumab included 138 patients with advanced solid tumors or B cell lymphoma. Urelumab demonstrated particular benefit in patients with melanoma: among checkpoint blockade-naïve melanoma patients, ORR was 50% and disease control rate was 70%, irrespective of PD-L1 status. Fatigue was the most frequent treatment-related AE (*n* = 43; 31%). Jennifer Wu, PhD (Medical University of South Carolina) closed the session with a presentation about the therapeutic potential of targeting soluble MHC I-chain related molecules (sMIC) in combination with immune checkpoint inhibition. Produced by tumors via proteolytic cleavage, sMIC has been shown to be highly immunosuppressive by binding and downregulating expression of its cognate ligand, NKG2D on NK and T cells. Preclinical animal models have demonstrated the feasibility of this approach as well as synergy with anti-CTLA-4 and anti-PD-1/L1 blockade.

### Presidential session

The Presidential Session featured outstanding presentations of the highest-ranking abstracts authored by young investigators. Each oral presentation was judged by an expert panel to determine the winner of the Presidential Award. This year, Roberta Zappasodi, PhD (Memorial Sloan Kettering Cancer Center) was awarded the Presidential Award for her work analyzing pharmacodynamic biomarkers in the first-in-human trial of GITR costimulation with the antibody agonist TRX-518. Pre-and post-treatment peripheral blood mononuclear cell (PBMC) samples were analyzed from 37 patients who received TRX-518 at increasing doses, along with pre- and post-therapy tumor biopsies from eight patients. Patients in the study had a variety of solid tumors, including melanoma (*n* = 6), NSCLC (*n* = 7), colorectal cancer (*n* = 7), and other solid tumors (*n* = 17). Among T cell populations analyzed, there was a marked reduction in circulating Treg in melanoma and colorectal cancer patients following treatment with TRX-518. Reflecting findings in the periphery, tumor biopsies from melanoma and colorectal cancer patients revealed that intratumoral FoxP3+ Treg cells were also reduced after GITR costimulation. These results identify circulating Treg as a potential biomarker of TRX-518 activity and warrant further investigation to determine a potential association with clinical response.

### Microbiome and the impact on local inflammation and host immunity

Romina Goldszmid, PhD (National Cancer Institute, National Institutes of Health) opened this session by presenting work demonstrating the ability of the gut microbiota to modulate the response to cancer therapy. Previous work established the adjuvant role and priming effect of gut microbiota in modulating the response to anti-cancer treatment, including both conventional and immune-targeted therapies [23, 24]. In order to elucidate the mechanisms underlying the role of the microbiota in modulating response to therapy, Dr. Goldszmid presented work characterizing the myeloid cell compartments in the TME as well as the bone marrow of germ-free and conventionally housed mice. These findings illustrated that the composition of the myeloid cell infiltrate was altered in germ-free mice both before and after treatment with oxaliplatin. Gene expression analysis also demonstrated marked differences in the cellular composition of germ-free mice. These results illustrate that the impact of microbiota on myeloid cells is important in understanding the mechanism of action of different tumor therapies and may contribute to differences in antitumor treatment response.

The influence of the microbiome on the efficacy of anti-cancer therapies was further elaborated by María Paula Roberti, PhD (Institute of Gustave Roussy). Dr. Roberti demonstrated that the absence of gut microbiota (naturally occurring in germ-free mice or induced by broad spectrum antibiotics [ATB]) compromised the anticancer activity of cyclophosphamide. Compensation with *Enterococcus hirae* not only restores the efficacy of cyclophosphamide lost with ATB by inducing pTh17 and Th1 responses, but also restores cyclophosphamide efficacy in germ-free mice by modulating the TME. The underlying mechanism involves disruption of the integrity of the intestinal epithelium, which promotes translocation of *E. hirae* in secondary lymphoid organs. NOD2 was identified as an important “gut immune checkpoint,” restricting the translocation and immunogenicity of *E. hirae* and inhibiting cyclophosphamide efficacy. The immunomodulatory role of gut microbiota on cancer therapeutics is also apparent with immune checkpoint blockade therapies, such as blockade of CTLA-4 by ipilimumab. Ipilimumab can modify the abundance of immunogenic *Bacteroides* spp. in the gut, which in turn impacts its anticancer efficacy. Uptake of distinct bacterial species or bacteria-derived products by DC in the context of immune checkpoint inhibition can significantly enhance DC antigen processing and presentation. This DC activation boosts the generation of antitumor T cells and increases intratumoral T cell numbers. These results suggest that modulating gut microbiota may represent a new therapeutic strategy to boost the antitumor efficacy of anticancer compounds.

### Tumor immunology 101 (nurse/pharm track)

Sessions dedicated to the new membership categories of nurse and pharmacist were featured this year, the purpose of which are to build up the foundational knowledge about cancer immunology and immunotherapy for the entire cancer care team. Beginning with a discussion about basic immunology for the non-specialist, Christian Capitini, MD (University of Wisconsin, Madison) described the major components of the immune system and the barriers to effective immunotherapy. In particular, Dr. Capitini explained how the innate and adaptive arms of the immune system work coordinately to generate an effective immune response as well as how tumors have figured out how to hijack these cells and create an immunosuppressive microenvironment that protects the tumor from elimination. Next, Satiro N. De Oliveira, MD (University of California Los Angeles) presented the basic principles of cancer immunotherapy using an illustration of the cancer immunity cycle to indicate where different types of cancer immunotherapy intervene to help drive the cycle toward eradication of the tumor. Emphasizing that the interplay between the immune system and malignant cells is a dynamic process, Dr. De Oliveira explained how combination therapies seeks to intervene at multiple stages in the cancer immunity cycle to limit the ability of the tumor to adapt and escape. Integrating the concepts presented throughout the session, Paul M. Sondel, MD, PhD (University of Wisconsin, Madison) closed with a forward-looking talk about the future of cancer immunotherapy, using some cutting-edge off-label examples. At present, highly engineered antibody-based therapeutics, chimeric antigen receptor (CAR) T cells, and combination approaches are currently used in different disease settings with increasingly positive patient outcomes and manageable associated toxicities. Immunotherapeutic strategies on the horizon will include combining different forms of immunotherapy, combining immunotherapies with conventional treatments, and moving toward personalized medicine by parsing out which patients should get which combinations at what time in their diagnosis.

### Clinical management (nurse/pharm track)

Following the introduction to tumor immunology, Kristin Kreamer, CRNP, MSN, AOCNP, APRN-BC (Fox Chase Cancer Center) delved into aspects of clinical management of immunotherapeutic agents, offering first a brief explanation of the CTLA-4 and PD-1/L1 pathways before providing an overview of immunotherapy agents currently approved for the treatment of melanoma, NSCLC, renal cell carcinoma, Hodgkin lymphoma, HNSCC, and bladder cancer. The next presentation, from Krista Rubin, MS, RN, FNP-BC (Massachusetts General Hospital), underscored the importance of prompt diagnosis and management of immune-related AE (irAE). This relies on understanding the mode of action of immune-based agents, which predicts toxicity and differentiates them from chemotherapy. Highlighting the most frequently encountered irAE (fatigue and dermatological, gastrointestinal, hepatic, and endocrine system dysfunction), Ms. Rubin proposed approaching symptoms with the adage, ‘it’s inflammatory until proven otherwise’. Toxicities are often reversible if addressed early, hence the value of offering patients a checklist of common symptoms as a resource. Using case studies, Brianna Hoffner, MSN, ANP-BC, AOCNP (University of Colorado, Denver) showed that endocrinopathies are less likely than other irAE to be reversible, hence the importance of early referral to the relevant disease area specialist. In the absence of consensus treatment algorithms, she recommended bringing patients back to the disease specialist’s clinic for management. Other key takeaways were the value of antibiotic prophylaxis to prevent infections during high-dose steroid use, and the need to taper steroids slowly; the free app for grading irAE; and the importance of considering the differential diagnosis for atypical symptoms. Long-term (often unusual) irAE can present for the first time even after long-term treatment discontinuation, so continued vigilance is essential.

### Diet, exercise, stress and the impact of the immune system

A new session about the effect of lifestyle on antitumor was hosted in collaboration with the Society of Behavioral Medicine. Dana H. Bovbjerg, PhD (University of Pittsburgh Cancer Institute) discussed new data on the immunosuppressive role of epinephrine, which is a catecholamine produced by autonomic nerves during stress responses. Epinephrine was observed to increase the suppressive cytokines IL-10 and IDO, mediated through the COX-2 axis. Macrophages stimulated with epinephrine prior to co-culture suppressed the proliferative and functional capacity of CD8+ T cells, an effect that could be reversed with the addition of the COX-2 inhibitor celecoxib. Further, tumor-associated macrophage production of IL-10 and IDO was shown to decrease following treatment with celecoxib.

Susan K. Lutgendorf, PhD (University of Iowa) also presented data on the effect of psychosocial stress on neuroendocrine function, inflammation, and tumor biology. Compelling data on how neural pathways associate with intrinsic tumor cell behavior, and specifically how stress signaling could promote tumor cell progression were shown. Patients with ovarian cancer who lacked social support and/or experienced more distress had reduced innate immunity and T cell responsiveness in the TME. Tumor analyses from those patients revealed a gene signature representative of enhanced tumor aggressiveness.

The effect of dietary restriction and exercise on tumor growth and metastasis in murine breast tumor models was discussed by Connie J. Rogers, PhD, MPH (Pennsylvania State University). Mice that maintained their body weight via mild dietary restriction (10% of calories) and daily exercise were shown to have a significant decrease in primary tumor growth and metastatic spread of 4 T1.2 mammary tumors. Moreover, the combination of diet and regular exercise significantly reduced the prevalence of immune suppressive MDSC and led to an enhanced response to vaccine immunotherapy. These data suggest that lifestyle interventions may improve responsiveness to emerging immunotherapies.

Graduate student Mark J. Bucsek (Roswell Park Cancer Institute) closed the session with data demonstrating that mice housed at the standard cool temperatures mandated for laboratory mice (~22 °C) provides a convenient tool for studying adrenergic stress and the immunosuppressive impact of norepinephrine through the β2-adrenergic receptor on CD8+ T cells. Reduction in β-AR signaling through elevated housing temperature or use of β-blockers improved the efficacy of anti-PD-1 therapy in tumor-bearing mice, compared to either monotherapy (both *p* < 0.001) and was associated with an increase in the number of IFNγ-producing CD8+ T cells.

### Adoptive cellular therapy vs. bispecific antibodies

Crystal L. Mackall, MD (Stanford University) introduced the session dedicated to forms of adoptive cellular therapy and bispecific antibody approaches with the observation that immune-based therapies for B cell malignancies have been on the leading edge of immunotherapy, and that these successes have provided opportunities to advance the larger field of cancer immunotherapy. Indeed, the first monoclonal antibody (rituximab, 1997), CAR T cell (CD19-CAR, 2010), and bispecific antibody (blinatumomab, 2011) therapies to demonstrate unequivocal antitumor activity were all in the setting of B cell malignancies. Among the factors that have allowed B cell malignancies to be on the cutting edge of immunotherapeutic advances include our relatively exquisite knowledge of the cell surface landscape of B cells as opposed to that of solid tumors. In addition, the tolerable off-tumor, on-target effects of targeting B cell malignancies make it an attractive candidate for immunotherapeutics. Although not yet formally demonstrated, it is speculated that the microenvironment of liquid tumors might be more permissive to immunotherapy than the immunosuppressive microenvironment of solid tumors. Dr. Mackall used this background to lead into a balanced presentation about which immunotherapy agent to use for the treatment of B cell malignancies: blinatumomab versus CD19-CAR T cells.

Importantly, no data from randomized controlled trials currently exist to indicate whether blinatumomab or CAR T cell therapy is the superior choice. This lack of data directly impacts the ability to evaluate response rates between the two therapeutic modalities. In general, and taking into account that very few of these trials are intent-to-treat trials, there are higher reported response rates in CD19-CAR single arm studies. In terms of durability of effect, blinatumomab has a very short half-life and clear data on whether blinatumomab leads to the acquisition of adaptive immunity have not yet been presented. The durability of CAR T cell responses is specific to which CAR T cell is used, as CAR.28.Z have been shown to persist 1–2 months whereas CAR.BB.Z persist 6–12 months. How well these agents can traffic into the tissues is another important consideration especially for acute lymphoblastic leukemia (ALL) where extra-medullary relapse can be a major issue. Although the tissue trafficking of blinatumomab is less clear, numerous groups have demonstrated that CAR T cells traffic very efficiently into the central nervous system. From a toxicity standpoint, there is no clear distinction between the CAR T cells and bispecific antibody therapies, as both are capable of inducing cytokine release syndrome, the severity of which is predominantly linked to the disease burden but can be managed safely in most cases.

As of 2016, the FDA has approved blinatumomab for adult and pediatric B cell ALL. Approvals for CD19-CAR T cells for both indications are anticipated in 2017. With these approvals, and as treating physicians develop more experience with these agents, patterns of clinical usage will emerge and larger studies will become available to inform treatment decisions. Some of the most important questions moving forward will be how best to incorporate these therapeutics into first- and second-line therapies, which is likely to require large cooperative group trials.

### Emerging technologies

In a session focused on recently emerging technologies, Sean G. Smith (University of North Carolina, Chapel Hill and North Carolina State University) presented results of a study utilizing novel intravesical immunotherapy to engage adoptive immunity in a murine model of bladder cancer. Composed of a coformulation of the biopolymer chitosan with interleukin-12 [25], CS/IL-12 was given intravesically twice a week for two weeks following orthotopic implantation of MB49 bladder cancer cells [26]. Survival was monitored following depletion of lymphocyte subtypes, and cellular responses were measured 24 h after each treatment via flow cytometry. Results illustrated that even a single treatment with CS/IL-2 extended survival in mice long-term after 1, 2, 3, or 4 treatments. Initial tumor elimination was found to be dependent on CD8+ T cells, while subsequent rejection after re-challenge was dependent on CD4+ T cells. Moreover, initial treatments were characterized by an increase in macrophages in the bladder and an increase in CD8:Treg ratio in the bladder-draining LN. By the third treatment, there was also an increase of CD4+ and CD8+ T cells in the bladder, with increased CD8+ T cells in the bladder-draining LN. These results illustrate the antitumor efficacy of this novel therapy and provide insight into the mechanisms of initial tumor rejection as well as memory response.

John-William Sidhom (Johns Hopkins University) presented a novel bioinformatics tool, ImmunoMap, to visualize and quantify TCR repertoire diversity utilizing a sequence analysis approach inspired by phylogenetics. Using tumor-bearing B6 mice, the utility of ImmunoMap was demonstrated by comparing the CD8+ T cell response to self (Kb-TRP2) and foreign (Kb-SIY) antigens. The tool was also applied to analysis of tumor infiltrating lymphocytes (TIL) in tumor biopsies from patients with metastatic melanoma enrolled in a clinical trial receiving nivolumab, in order to compare the TCR repertoire of responders vs. non-responders. Analysis of CD8+ T cell response to SIY illustrated lower clonality, with TCR that were structurally similar. In contrast, response to TRP2 showed CD8+ T cells that were highly clonal but less structurally related, which may reflect effects of peripheral tolerance on self vs. foreign antigens. Clinical trial data showed that unique TCR signatures differentiated nivolumab responders from non-responders. Moreover, some of these signatures could be detected prior to therapy. ImmunoMap revealed that immunotherapy responders had a pre-existing repertoire that was more structurally diverse prior to therapy but became less diverse during therapy. Taken together, this analysis indicates that patients with a broader T cell repertoire prior to therapy have a higher probability of expanding effective TCR sequences and converging on them. This new tool may enable quantification of TCR repertoire diversity from complex sequencing analyses and may also facilitate identification of predictive biomarker signatures.

## Conclusions

SITC’s 31st annual meeting was replete with novel data and strategies for improving cancer immunotherapeutics. The largest annual meeting to date, SITC 2016 continued the tradition of facilitating the collaboration between researchers and oncology health care professionals that is essential for moving immunotherapy into the future. Ongoing efforts to drive advancements in the field are focused on increasing the number of responding patients via a better understanding of the mechanisms by which current cancer immunotherapeutic agents function, identifying predictive and prognostic biomarkers, developing rational combination therapies, and recognizing and managing irAE. Join SITC for the 32nd Annual Meeting and Pre-Conference Programs, which will take place November 8–12, 2017 in National Harbor, Maryland.

## Abbreviations

AE: Adverse event (s)
AFP: Alpha fetoprotein
ALL: Acute lymphoblastic leukemia
AML: Acute myeloid leukemia
CAR: Chimeric antigen receptor
CI: Confidence interval
CITN: Cancer immunotherapy trials network
CTLA-4: Cytotoxic T lymphocyte-associated protein 4
CVA21: Coxsackievirus A21
DC: Dendritic cell (s)
DCB: Division of cancer biology
DCTD: Division of cancer treatment and diagnosis
FDA: U.S. food and drug administration
FRET: Fluorescence energy resonance transfer
HCC: Hepatocellular carcinoma
HNSCC: Head and neck squamous cell carcinoma
IDO: Indoleamine 2, 3-dioxygenase
IFN: Interferon
irAE: Immune-related adverse event (s)
JITC: Journal for immunotherapy of cancer
KIR: Killer immunoglobulin-like receptor (s)
LN: Lymph node (s)
MDSC: Myeloid-derived suppressor cell (s)
MSI: Microsatellite instability
NCI: National cancer institute
NE: Norepinephrine
NIH: National institutes of health
NIMBC: Non-muscle invasive bladder cancer
NK cell: Natural killer cell
NR: Not reached
Nrp1: Neuropilin-1
NSCLC: Non-small cell lung cancer
OCE: Oncology center of excellence
OS: Overall survival
PBMC: Peripheral blood mononuclear cell (s)
PD-1: Programmed cell death protein 1
PD-L1: Programmed death-ligand 1
PFS: Progression-free survival
SITC: Society for immunotherapy of cancer
TCR: T cell receptor
TIL: Tumor infiltrating lymphocyte (s)
TLR: Toll-like receptor
TME: Tumor microenvironment
Treg: Regulatory T cell (s)
WG: Working group (s)
β-AR: β-adrenergic receptor
